# Core Cognitive Mechanisms Underlying Syntactic Priming: A Comparison of Three Alternative Models

**DOI:** 10.3389/fpsyg.2021.662345

**Published:** 2021-06-28

**Authors:** Yuxue C. Yang, Ann Marie Karmol, Andrea Stocco

**Affiliations:** Cognition and Cortical Dynamics Laboratory, Department of Psychology, University of Washington, Seattle, WA, United States

**Keywords:** syntactic priming, declarative knowledge, procedural knowledge, reinforcement learning, computational modeling

## Abstract

Syntactic priming (SP) is the effect by which, in a dialogue, the current speaker tends to re-use the syntactic constructs of the previous speakers. SP has been used as a window into the nature of syntactic representations within and across languages. Because of its importance, it is crucial to understand the mechanisms behind it. Currently, two competing theories exist. According to the transient activation account, SP is driven by the re-activation of declarative memory structures that encode structures. According to the error-based implicit learning account, SP is driven by prediction errors while processing sentences. By integrating both transient activation and associative learning, Reitter et al.'s hybrid model 2011 assumes that SP is achieved by both mechanisms, and predicts a priming enhancement for rare or unusual constructions. Finally, a recently proposed account, the reinforcement learning account, claims that SP driven by the successful application of procedural knowledge will be reversed when the prime sentence includes grammatical errors. These theories make different assumptions about the representation of syntactic rules (declarative vs. procedural) and the nature of the mechanism that drives priming (frequency and repetition, attention, and feedback signals, respectively). To distinguish between these theories, they were all implemented as computational models in the ACT-R cognitive architecture, and their specific predictions were examined through grid-search computer simulations. Two experiments were then carried out to empirically test the central prediction of each theory as well as the individual fits of each participant's responses to different parameterizations of each model. The first experiment produced results that were best explained by the associative account, but could also be accounted for by a modified reinforcement model with a different parsing algorithm. The second experiment, whose stimuli were designed to avoid the parsing ambiguity of the first, produced somewhat weaker effects. Its results, however, were also best predicted by the model implementing the associative account. We conclude that the data overall points to SP being due to prediction violations that direct attentional resources, in turn suggesting a declarative rather than a RL based procedural representation of syntactic rules.

## Introduction

Syntactic Priming (SP, also known as “Structure Priming”) is the linguistic phenomenon by which speakers tend to reuse syntactic structures across utterances (Bock, 1996). Its existence is often touted as the strongest evidence that the same syntactic mechanisms are used in both language comprehension and language production. As such, manipulations that affect SP can be used to gather insight into how the brain perceives, represents, and applies syntactic structures. For example, two notable studies (Loebell and Bock, 2003; Hartsuiker et al., 2004) have shown that SP effects occur across languages, demonstrating that syntactic structure is represented in a way that is language independent.

In this study, we use the syntactic priming paradigm to better understand the possible cognitive mechanisms underlying syntactic representation. Consistent with an emergentist approach to language (Hernandez et al., 2019), we hypothesized that the syntactic operations are not modular and encapsulated but depend on general-purpose cognitive mechanisms, such as procedural learning or working memory [“a new machine built out of old parts,” as Bates and Benigni (1979), famously put it].

To identify which cognitive functions, specifically, support syntax in SP effects, we devised a variation of the SP paradigm that includes a new critical manipulation, that is, the presence of syntactically incorrect priming sentences. As it will be shown, different existing theories of SP and different. These predictions were tested in two different experiments.

### Existing Theories of Syntactic Priming

In the past few decades, many researchers have attempted to determine the most likely mechanistic explanation for SP (Hartsuiker et al., 2004; Chang et al., 2006; Reitter et al., 2011). Experimental studies showed that a range of factors could impact the strength of priming. For example, the priming effect is enhanced by the presentation of multiple primes, which is referred to as the cumulativity of SP (Jaeger and Snider, 2008). In addition, the lexical overlapping between prime and target also enhances priming, which is known as the lexical boosting effect (Pickering and Branigan, 1998). Moreover, there is evidence for an inverse frequency interaction, showing that the less frequently used syntactic structures are associated with stronger priming effects (Bock, 1986; Jaeger and Snider, 2008; Kaschak et al., 2011).

Several competing accounts have been put forward to explain these effects. A group of researchers, for example, advocated a transient short-term residual activation account relying on a declarative system (Pickering and Branigan, 1998; Branigan et al., 2000; Pickering and Garrod, 2004; Kaschak, 2007), which argues that the increased probability of using a syntactic construction depends on how frequently it has been retrieved from the memory. Another group of influential accounts are built upon implicit learning theory, assuming the processing of a syntactic structure affects the structure's probability distribution (Bock and Griffin, 2000; Chang et al., 2006).

A syntactic structure's probability distribution can be learned in multiple ways. For example, Chang et al. (2006) presented a connectionist model in which syntactic acquisition is a consequence of error-based implicit learning. In language processing, the deviation between one's prediction and observed information (that is, the prediction error) serves as a learning signal, and the weights in the network are updated in order to minimize the prediction error. Because the prediction error naturally leads to implicitly learning the statistics of occurrence of different syntactic structures, it provides an elegant explanation for the cumulative effect.

Jaeger and Snider (2008) built upon this idea and proposed a surprise-sensitive persistence account, which is able to explain the inverse frequency effect. Their account assumes that syntactic priming is caused by updating and maintaining the probability distribution of syntactic information. Specifically, this account predicts that more surprising syntactic structures (lower frequency), lead to greater change of the prior probability distribution, and thus lead to activation boosts of this particular structure, enhancing the priming effect by increasing the probability of reusing this construction.

Reitter et al. (2011) presented a hybrid model, in which syntactic priming is achieved through a combination of frequency-driven boosts of activation from transient processing (Pickering and Branigan, 1998) and contextual associations that drive predictions (Chang et al., 2006). In Reitter's et al. hybrid model, the probability of certain syntactic structures being produced depends on its activation relative to other syntactic structures in memory. A structure's activation follows the rules of memory decay, thus exhibiting frequency and recency. Contextual associations further boost a structure's base activation. Reitter's et al. model predicts inverse frequency effect by arguing that most recent exposure of syntactic structure increases its base activation, leading to higher probability of being retrieved with retrieval cue. Specifically, less frequent constructions have relatively lower base activation compared to more frequent ones and thus leads to a larger relative increase in activation boost caused by processing prime construction.

### Connecting Syntactic Priming to Core Cognitive Mechanisms

The goal of this paper is to connect these possible accounts of SP to existing and general mechanisms that might exist in the brain (Hasson et al., 2018). For example, there are multiple mechanisms that could be used to implement a prediction error or surprise-based learning of probability distributions about syntactic structures. Ultimately, the choice of the specific mechanisms is tied to the specific way in which syntactic knowledge is believed to be represented and its putative neural substrate.

Perhaps the most general distinction that can be made is between declarative and procedural knowledge (Knowlton and Squire, 1994; Squire, 2004). Declarative knowledge, which encompasses episodic and semantic memory, possesses many of the properties that are assumed to be shared by syntactic structures. In particular, its availability reflects the frequency with which a particular item has been processed. It also decays over time, with the transient boost of activation giving rise to priming effects. Note that, although declarative knowledge is typically explicit, the mechanisms that regulate its availability (frequency, recency, spacing, and priming) remain implicit and thus compatible with the assumptions of existing models. A declarative account is also compatible with the findings of Ivanova et al. (2012), which ultimately point to a lexicon-based nature of syntactic priming effects—the mental lexicon is vastly believed to be represented in declarative (semantic) memory. Reitter et al. influential model 2011 of syntactic priming, for example, relies entirely on declarative representations.

However, syntactic structures could also be potentially represented as procedural knowledge. In Ullman's declarative-procedural model (2004), for example, syntactic structures are explicitly identified with procedural knowledge. Procedural knowledge is considered intrinsically implicit and non-verbalizable, making it naturally compatible with implicit learning accounts. Since procedural knowledge is used to represent “how-to” information, it naturally leads to the complex operations of syntactic rules. Unlike declarative knowledge, which is known to be reflect frequency and recency, procedural knowledge is thought to be refined through reinforcement, and specifically by reward prediction errors (Sutton and Barto, 1998). Rules and operations that are most commonly successful are believed to be applied more frequency; violations of these expectations are known to drive learning. Thus, the learning mechanisms of procedural knowledge also rely on frequency and prediction violations, and are in principle compatible with syntactic priming effects.

Despite their similarity, it is possible, albeit difficult, to distinguish between declarative and procedural representations. For example, Anderson et al. (1997) relied on the fact that procedural knowledge, being habitual, is less flexible and more difficult to apply in uncommon orders. Jacoby (1991) also demonstrated that, since procedural knowledge is implicit, participants cannot successfully control or prevent its application. Stocco and Fum (2008) showed that, since the use of procedural knowledge is shaped by previous rewards, it is difficult to prevent its application in circumstances where it would lead to negative outcomes.

Most psycholinguistic studies investigated syntactic priming effects using carefully controlled experimental items, ensuring that the linguistic stimuli have no mistakes and are produced flawlessly. However, in natural conversation, disfluencies and errors are very common when people are speaking. Usually, speech errors (which might include ungrammatical constructions, inappropriate word choices, ambiguous meaning, or absolute non-sense) are considered as interference that either slows down the processing or impedes comprehension. Even though people may ignore minor speech errors in daily conversation, there is evidence that erroneous information does affect language processing, and might provide a further cue to the underlying representation of syntax. For example, people often change their mind and correct themselves mid-sentence while speaking. Slevc and Ferreira (2013) examined the priming effect in the context of correcting speech errors. They found that SP is significantly reduced when primes were corrected to the alternative syntactic structure.

Another study by Ivanova et al. (2012) investigated whether people tend to orally produce ungrammatical utterances by immediate exposure to ungrammatical primes—that is, if syntactic priming extends to ungrammatical constructs as well. They compared two competing accounts of syntactic priming, abstract structural persistence account, which argues that structural priming occurs because of the availability of an abstract rule; and a lexically driven persistence account, which argues that only the exposure to the exact same lexical elements leads to ungrammatical priming. They specifically looked at when the target ungrammatical verb-construction would occur in participants' speech by manipulating the syntactic structure of primes. For example, the sentence “The dancer donates the soldier the apple” is a grammatically incorrect sentence because in standard English, the verb “donate” does not permit the dative alternation. According to the abstract structural persistence account, reading the same double-object constructions, such as “The waitress gives the monk the book” would lead participants to produce the same verb-construction combinations even though the utterances tend to be ungrammatical. On the contrary, the lexically driven persistence account predicts that the priming only occurred if the lexically-specific syntactic information, the exact verb “donate” is repeated in both prime and targets. Their findings supported lexically-driven persistence account, against abstract structural persistence account, showing that no structural priming effect of ungrammatical double object responses was found when the prime shared similar structure but not exact lexical information.

This raises a question related to our research interests, in written production, whether the SP effects would be different if the prime contains grammatical errors and which account, activation boosts, associative learning, or reinforcement learning, is the best to account for our findings. In this study, we proposed three hypothetical models depending on three accounts discussed above, and attempted to account for an ungrammatical priming pattern by comparing them. First, an transient activation account assumes that the SP is the result of memory retrieval. The error which violates the grammatical rules is not expected to be parsed in the priming process, thus according to this account, there should be no change in SP effects by introducing grammar errors. The second account is based on Reitter et al. model 2011, which argues that SP involves both transient activation boosts and associative learning. Like error-based implicit learning, this account consistently predicts that the rare a construction is, the stronger the priming is (Jaeger and Snider, 2008, 2013; Reitter et al., 2011). Given that the ungrammatical constructions are less commonly seen, we assume that there is a possibility that the SP could be enhanced by grammar errors. In addition, the role played by errors in SP introduces a third point of view on the nature of SP, which can be cataloged under the RL account. According to this point of view, syntactic structures are represented procedurally and their selection is guided by their perceived utility in terms of Reinforcement Learning, i.e., their estimated future amount of “rewards” or positive feedback signals (Sutton and Barto, 1998). It is widely accepted that procedural knowledge, in general, is refined in a Reinforcement Learning-like manner through the backpropagation of reward or feedback signals. In fact, procedural knowledge and reward signals share the same computational substrate, in the dopaminergic basal ganglia (Schultz et al., 1997; Yin and Knowlton, 2006). Furthermore, although the basal ganglia are not considered part of the cortical language network, an increasing number of studies have shown their involvement in language processing (Friederici, 2006; Stocco et al., 2014).

### Present Study

In this study, we set forward to test different accounts for syntactic priming, and to answer the question of whether perceiving incorrect linguistic information such as ungrammatical syntactic constructions would affect people's subsequent language representation, particularly in syntactic choices of production. In Experiment 1, we used Active/Passive primes with grammar errors to examine the change of syntactic production preferences. Then we attempted to explain the observed patterns by fitting the empirical results in the ACT-R model simulations. In Experiment 2 we changed to another extensively studied syntactic structure, double-object dative(DO)/prepositional-dative(PD) constructions, and manipulated the position of grammar error to investigate whether the priming pattern could be accounted for by three models.

### Theoretical Hypothesis

Based on discussed theories of SP, we apply similar principles to compare three hypothetical models that account for priming effects using different combinations of declarative and procedural mechanisms. Specifically, we argue that procedural vs. declarative accounts of syntax can be distinguished by how syntactic priming is modulated by ungrammatical prime sentences.

Across all predictions, we expected that syntactic priming effects would occur regardless of syntactic correctness. Specifically, the proportion of producing the same construction was expected to be higher than producing alternative construction. We also expected that the priming effect would be different depending on whether the syntactic structure of prime was correct or not.

The Activation model assumes that the syntactic structures are represented in declarative knowledge, and that syntactic priming effects depend on the frequency and recency of syntactic structures that are encountered. This model implements the majority of mechanics of Reitter et al. model 2011 except the associative spreading component. In this model, the error which violates the grammatical rules is not expected to be parsed in the priming process, thus according to this account, there should be no change in SP effects by introducing grammar errors.

The Associative model is based on Reitter et al. hybrid model 2011, in which syntactic structures are also represented declaratively, but additional activation is also provided during processing through associative links. In this model, ungrammatical primes require additional processing and, because of these additional resource demands, gather additional boost of spreading activation. In this model, thus, ungrammatical sentences further amplify the syntactic priming effect.

Finally, In the Reinforcement model, syntactic structures are represented procedurally and their selection is guided by their perceived value in terms of reinforcement learning, i.e., their predicted future positive feedback signals (Sutton and Barto, 1998). In this model, procedural rules compete to process sentences, and are reinforced by successes. As discussed, reinforcement learning mechanisms also reflect frequency and expectations, and are thus capable of replicating the main syntactic priming findings. Since ungrammatical sentences do not match the expected syntax, however, they are likely to result in a negative rather than a positive feedback signal. Thus, according to the model, ungrammatical sentences would dampen or reverse, rather than amplify, the syntactic priming effect.

## Computational Modeling in ACT-R

To explicitly formulate the three hypotheses, we implemented them as three different computational models. Each model performs a simplified version of a canonical SP task, first comprehending a sentence (in either active or passive form) and then producing a sentence to describe a picture. Both comprehension and production depend on the use of two rules that implement the active and the passive sentence structures. In comprehension, these rules are used to mediate from the underlying sentence to its higher-level semantic representation. In language production, these rules are used to create a mental plan of the sequence of words to produce a description of the picture. The crucial difference among three models is how the rules are represented and what is the cause of the priming, that is, the transient increase in probability of using a particular syntactic rule after encountering it. A summary of these differences is given in Table 1.

**Table 1:** Overview of the differences between models.

**Models**	**Rule representation**	**Priming mechanisms**
Activation model	Declarative memory	Recency
Associative model	Base-Level learning and associative learning	Spreading activation
Reinforcement model	Procedural memory	Feedback signal

All three models were implemented in ACT-R (Anderson et al., 2004), which is the dominant cognitive architecture in psychology and neuroscience (Kotseruba and Tsotsos, 2020). As a cognitive architecture, ACT-R provides a series of basic cognitive functions that synthesize that current understanding of cognitive and neural computations. By implementing all three models in the same architecture, we are ensuring that the three models reflect the exact mechanisms and that parameters that occur in more than one model have the same effect and interpretation.

Although a full overview of ACT-R is beyond the scope of this study, it is important to highlight some general characteristics of ACT-R that are important for our models. ACT-R models have two types of long-term memory representations, declarative and procedural. Declarative memory is stored in vector-like structures called *chunks*, which are used to represent semantic and episodic memories (“Paris is the capital of France”), perceptual inputs (“A black triangle is on the screen”), or motor commands (“Press the spacebar”). Chunks have an associated scalar quantity, activation, that represents the probability of a chunk to be retrieved at any given time; this probability decays exponentially over time and increments after every retrieval of the chunk, capturing both recency and frequency effects. Thus, activation reflects the transient increases in the availability of a piece of information as it is processed again.

Procedural knowledge is stored in conditional state–action rules, called production rules or *productions*, that encode basic stimulus-response associations (“if you hear the bell, prepare for food”), habits (“if you go out, pick up an umbrella”), and minimal mental steps (“if you attend to something, place it in working memory”). At any time, multiple productions might be available and competing for execution; the winning one is determined on the basis of their *utility*, a scalar quantity that reflects the probability of a particular rule to generate rewards and is learned through a temporal-difference reinforcement learning algorithm (Sutton and Barto, 1998), implemented as in Equation (3). Just like reprocessing of information causes a transient surge in the availability of the corresponding chunks, so the successful application of a rule causes a transient increase in its utility of the corresponding production.

Chunks are accessible to production rules via a set of dedicated, limited-capacity *buffers*. Buffers represent functionally specific cortical regions; for example, the retrieval buffer holds chunks retrieved from long-term memory and represents the function of the lateral prefrontal cortex (Anderson et al., 2008). Buffers also have an associated scalar value, *spreading activation*, which is thought to reflect the deployment of attentional resources (Daily et al., 2001). Activation spreads from chunks in buffers through all chunks in long-term memory that are associated (or share features) with them. These associations can be learned in a way that resembles Hebbian learning and provide another means to temporarily alter the probability that a chunk will be retrieved. The effect of spreading activation is modeled as an additional term added to each chunk's base-level association.

In summary, in the ACT-R modeling framework, cognition unfolds as productions respond to stimuli and changing mental states by retrieving, placing, and modifying chunks in the buffers, which in turns alterns both the probability of chunks to be retrieved and which production will be firing next.

### Model Design

Although ACT-R provides a general framework for modeling cognition, when modeling specific processes researchers often make different assumptions about the format of the underlying representations. For example, interference in the Stroop task can be modeled using either declarative (van Maanen et al., 2009) or procedural knowledge (Lovett, 2005). The same principle holds for language processes, with some authors representing syntactic structure as procedural rules (Lewis and Vasishth, 2005) and others representing them in declarative memory chunks (Stocco and Crescentini, 2005; Reitter et al., 2011). This grants us the possibility to explore syntactic priming as either a procedural or a declarative phenomenon.

To generate three different models of SP, each of which captures one proposed explanation of the SP effect and each of which depends on a single mechanism. All of these models can perform a rudimentary and highly stylized version of language understanding and production, and thus can perform the basic two steps of a SP task, that is, understanding a sentence describing a picture and composing a sentence to describe one.

The Activation Model relies on a declarative module to retrieve memory of syntactic structure. First, the model parses in a prime sentence from the visual buffer, and requests a retrieval of a syntactic structure based on what has been parsed in the imaginal buffer. After successfully retrieving the syntactic structure (or failing to retrieve), the model proceeds to the language production task, harvesting the target picture from the visual buffer and encoding it in the imaginal buffer. Then the model requests a retrieval of any available syntactic structure. ACT-R uses a base-level learning function to calculate the activation of chunks when a retrieval request is made (Equation 1). The activation of the syntactic structure chunk reflects the degree to which prior experiences and current context, and determines whether it will be retrieved or not. The chunk with the greatest activation will be placed into the retrieval buffer. Given the retrieval outcomes, the model produces the sentence by applying the corresponding syntactic structure to the outcome sentence. If it fails to retrieve any syntactic structure from the declarative module, an “unknown” output will be generated.

$$\begin{array}{l} A_{i\;}=B_{i}\;+\;\varepsilon =\;\ln\;\left(\overset{n}{\underset{j=1}{\sum }}{t_{j}}^{-d}\right)\;+\varepsilon \end{array}$$

**Equation 1**. Base-learning activation. Activation *A*_*i*_ consists of two main components: base-level activation *B*_*i*_ which reflects the recency and frequency of practice of chunk *i*; and a noise component ε. *n* indicates the number of presentations for chunk *i*, *t*_*j*_ is the time since *jth* presentation, *d* is the decay parameter.

The Associative Model implements associative learning in ACT-R, which accounts for the ungrammatical priming effects by including a context component in chunk activation (Equation 2). As a grammar error is parsed in, the chunk carrying this specific syntax information will be placed in the imaginal buffer, becoming the source of activation. This chunk can spread an amount of activations to chunks in declarative memory, resulting in higher likelihood of this syntactic structure being retrieved in the future (Equation 2).

$$\begin{array}{l} A_{i\;}=B_{i}\;+\;\underset{k}{\sum }\underset{j}{\sum }W_{kj}S_{ji}\;+\varepsilon =\;\ln\;\left(\overset{n}{\underset{j=1}{\sum }}{t_{j}}^{-d}\right)\\ \;+\;\underset{j}{\sum }W_{j}\left(S-\ln\left(\;fan_{j}\right)\right)+\varepsilon \end{array}$$

**Equation 2**. Activation *A*_*i*_ consists of three main components: base-level activation *B*_*i*_, context component, and a noise component ε. *W*_*kj*_ indicates the amount of activation from source *j* in buffer *k*, *S*_*ji*_ is the strength of association from source *j* to chunk *i*, *fan*_*j*_ is the number of chunks in declarative memory in which *j* is the value of a slot plus one for chunk *j* being associated with itself.

The Reinforcement Model uses procedural knowledge to represent grammatical rules, and reinforcement learning to select between competing rules. It first parses in a prime sentence from the visual buffer and creates a mental representation in the imaginal buffer. Feedback signals are generated by detecting whether the comprehended sentence is grammatically correct or not and are delivered at the end of the comprehension process. When the model proceeds to picture description tasks, two productions of syntactic structure compete with each other. The one with higher utility is chosen by the model to apply corresponding syntax. Equation (3) demonstrates the utility calculation equation.

$$\begin{array}{l} U_{t}\;=\;U_{t-1}\;+\;\alpha \left(R_{t}\;-\;U_{t-1}\right) \end{array}$$

**Equation 3**. Utility Learning in Reinforcement Learning. *U*_*t*_ represents the utility *U* of the production p at time point *t*, α indicates the learning rate, *R*_*t*_ is the reward the production received for at time *t*.

### Model Evaluation

In model selection, it is common to use likelihood-based measures. The likelihood function of a particular model with parameters θ, *L*(*m*, θ| *x*), is the probability that, given the parameterized model and set of observed data to fit, the model would produce that data = *L*(*m*, θ| *x*) = *P*(*x*|*m*, θ). Here, *m*and θrefers to the model and its parameters, and *x* refers to the observations. Common comparison metrics, such as the Akaike Information Criterion (AIC) and the Bayesian Information Criterion (BIC), are both based on likelihood. The problem is that, while closed-form likelihood functions have been derived for simple models (such as logistic models or linear models), they can be incredibly difficult to derive for relatively complex models and impossible for arbitrarily complex models based on the ACT-R architectures. In turn, this discourages the use of modern model selection procedures. Some attempts have been made. For example, both Stocco (2018) and Haile et al. (2020) used BIC to compare competing ACT-R models. However, the equation used to estimate BIC is a closed-form approximation that is based on Residual Sum of Squares and was originally derived for linear models; as such, it does not necessarily hold for ACT-R.

In this paper, we followed the computationally expensive but more accurate solution of empirically calculating the likelihood function but simulating each model and set of parameters multiple times, and calculating the empirical probability distribution of each results. Knowing the mean and standard deviation of this distribution, the value of *P*(*x*| *m*, θ) can then be calculated directly. If a model is designed to predict *n* data points (corresponding, for instance, to different experimental conditions), its likelihood can be expressed as the joint probability that any of those data points can be produced. For simplicity, and assuming independence, this can be expressed as the product of the probability of observing each individual data point in the empirical data, i.e., *L*(*m*, θ | *x*_1_, *x*„ … *x*_*n*_) = Π_*i*_ *L*(*m*, θ | *x*_*i*_,). Finally, to avoid computational problems with vanishing small probabilities, it is common the express this value in terms of *log* likelihood:

$$\begin{array}{l} \log L\;=\;\log\;P\left(x\;|m,\theta \right)=\;\underset{i}{\sum }\log\;z\left(x_{i}\;-x_{i,m}\right)\;/\;\sigma_{i,m} \end{array}$$

**Equation 4**. Log-likelihood of model selection. log*L* refers to the log of probability of observation *x* given model, *m* and parameters θ. *z* indicates the z-transformation, *x*_*m*_ indicates model outputs, σ_*m*_ indicates the standard deviation of model outputs.

### Model Fitting

**Figure 1:**
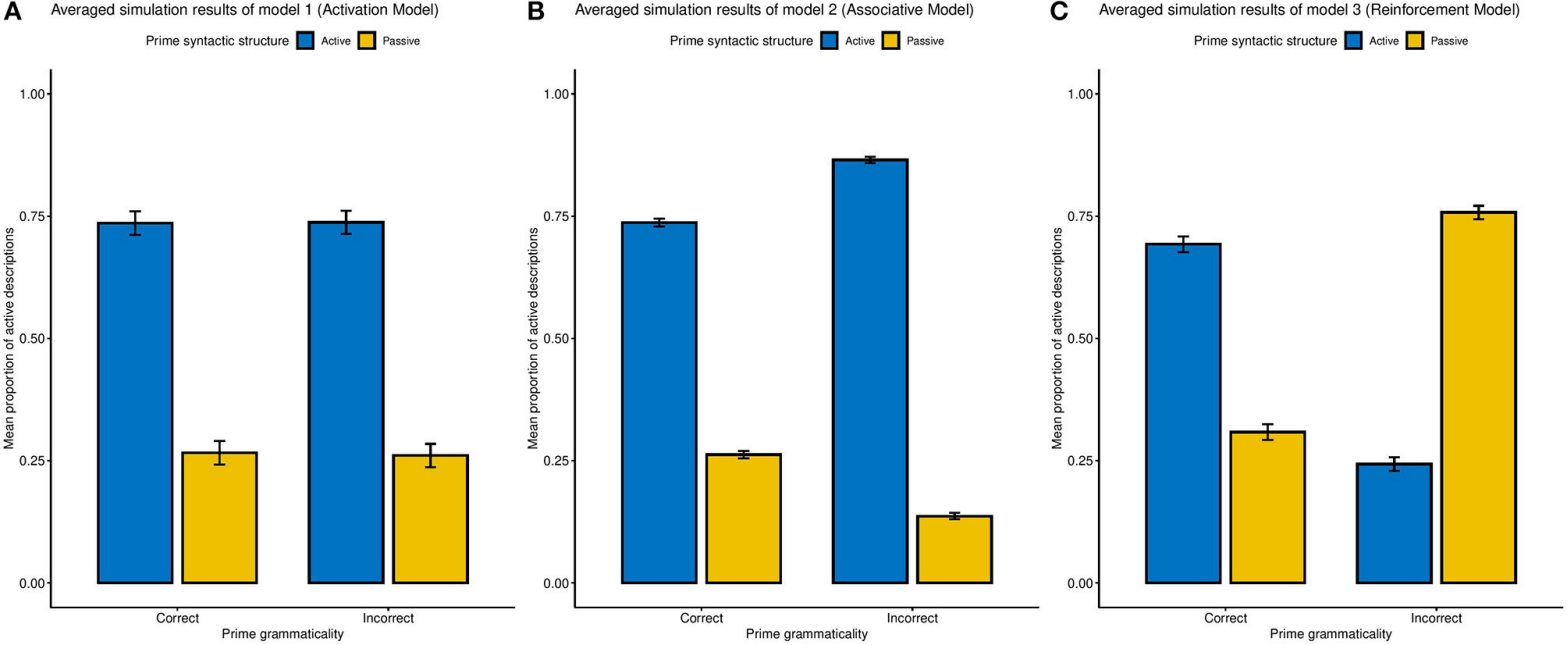
Averaged simulation results from three hypothetical models of SP effect across all parameter sets, with error bars representing the SD of simulation outputs. **(A)** The Activation Model (Model 1). **(B)** The Associative Model (Model 2). **(C)** The Reinforcement Learning model (Model 3).

To examine the predictions of our model, we use a grid-search approach to find the best possible parameters and the parameter space for each model is displayed in Table 2 (as in Haile et al., 2020). Each model simulates 40 independent trials the same as the experimental paradigm used for participants, running repeatedly for 50 times. Following the four different prime sentences, the mean proportion of each syntactic construction and their standard deviations are computed. We find that, across parameters, the three models reliably produce the qualitative pattern of our three hypotheses (Figure 1). Specifically, the simulations show that, in the Activation Model, the proportion of Active constructions does not change with respect to the grammar error in primes; that, in the Associative Model we observed a diminished SP effect after grammatically in correct constructions; and that in the Reinforcement Model the incorrect primes generate an enhanced SP effect.

**Table 2:** Model parameters manipulated in the simulations.

**Models**	**Parameter**	**Value**	**Meaning**
Declarative model	:ans	0.1, 0.25, 0.5, 0.75, 1, 1.5	Instantaneous noise
	:bll	0.1, 0.3, 0.5, 0.7, 0.9	Decay parameter in base-level learning
	:lf	0.1, 0.3, 0.5, 0.7, 0.9	Latency factor
Spreading model	:ans	0.1, 0.25, 0.5, 0.75, 1, 1.5	Instantaneous noise
	:bll	0.1, 0.3, 0.5, 0.7, 0.9	Decay parameter in base-level learning
	:lf	0.5, 0.7, 0.9, 1	Latency factor
	:ga	0.5, 1, 1.5, 2	Spreading activation parameter for goal buffer
	:mas	2.8, 3.2, 3.6	Maximum associative strength
Reinforcement model	:egs	0.01, 0.1, 0.5, 0.9, 1.3	Utility noise
	:alpha	0.1, 0.3, 0.5, 0.7, 0.9	Learning rate
	:r+	0, 0.1, 0.5, 1, 5, 10	Positive reward
	:r-	−10, −5, −1, −0.5, −0.1, 0	Punishment (negative reward)

In order to evaluate the three models for each participant, we used the Bayesian Information Criterion (BIC; Schwarz, 1978). The BIC balances fit and complexity by a penalty to the likelihood that is proportional to the number of parameters in a model. The relative value of BIC is more important than absolute BIC in interpreting the model performance. Low BIC indicates high likelihood of the model being able to fit participant data compared to other models, when a model's inherent complexity is taken into account. Specifically, the BIC can be calculated for each model as such (Equation 5):

$$\begin{array}{l} BIC\;=\;-\;2\;\log L\;+\;k\;\log\left(n\right) \end{array}$$

**Equation 5**. BIC estimation equation. *LogL* is the log-likelihood of model, *k* is the number of parameters, *n* is the number of observations.

### Model Comparison

Finally, to make group-level inferences from this pattern of variability, we used a Group Bayes Factor (GBF) approach (see, for example, Stephan et al., 2007). Group-level likelihood values for a model *m* can then expressed as the product of the likelihood of that model fitting the specific results *x*of each participant *p*, i.e., Π_*p*_ L(*m*, θ | *x*_*p*_). When using log-likelihood, this translates to the sum of all of the participant's log-likelihoods ∑ _*p*_ log L(*m*, θ | *x*_*p*_). The GBF is then computed as the ratio of the group likelihoods of two models, *L*(*m*_1_, θ | *x*_*p*_)/*L*(*m*_2_, θ | *x*_*p*_). In terms of log-likelihood, the GBF can be expressed as e^*d*^, with *d* being the difference in log-likelihoods between the two models. Kass and Raftery (1995) provide guidelines to interpret these values.

## Experiment 1

**Figure 2:**
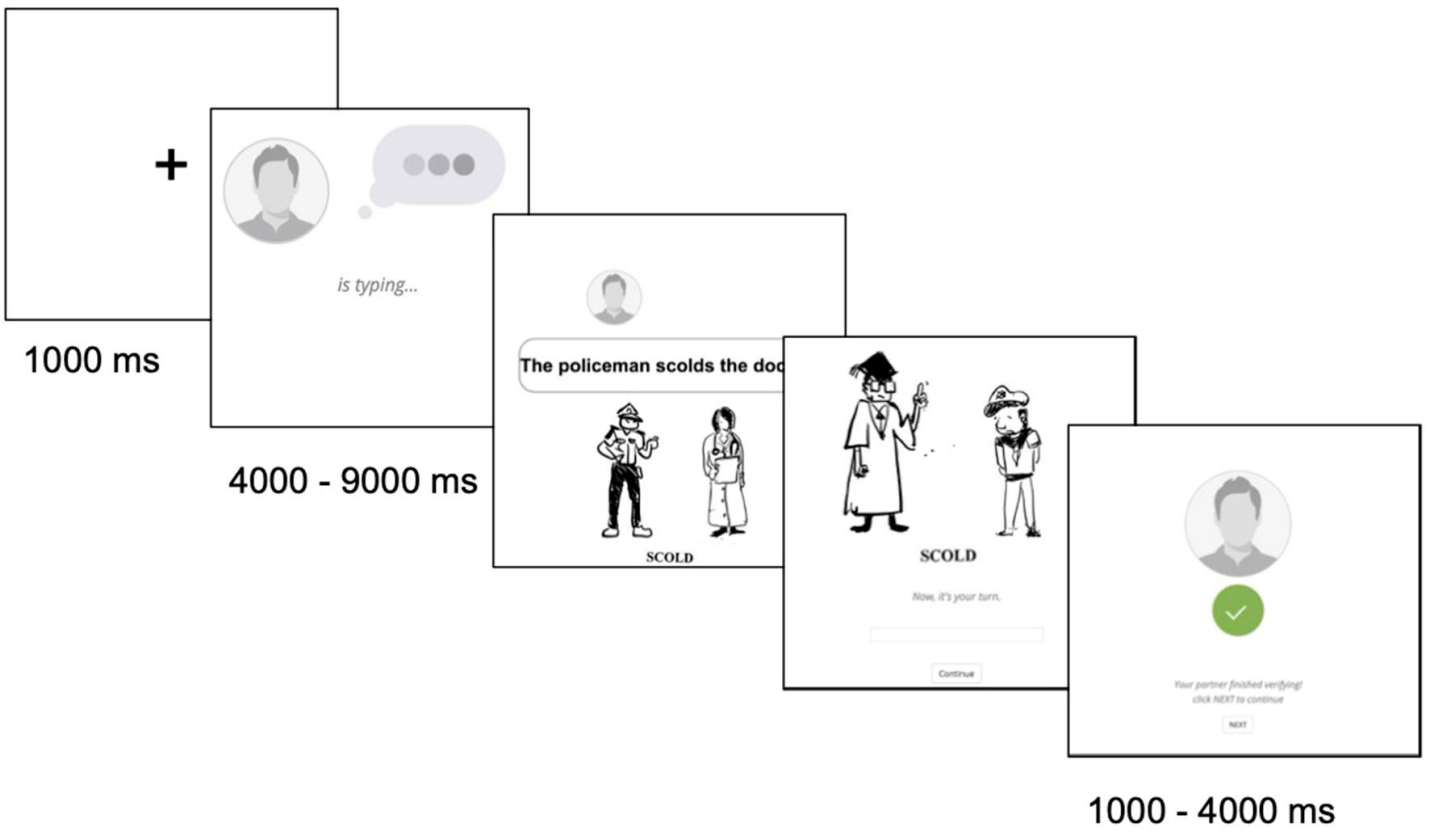
An example trial of Experiment 1, including a verification task and a description task. Verification task: an online “partner” (confederate) was typing a sentence to describe the picture shown below. Participants needed to verify whether the sentence and the picture matches. Picture description task: participants typed a sentence to describe the picture and waited for the “partner” to verify the response. The picture stimulus is only for demonstration purpose, not the real ones used in the study.

In Experiment 1, participants completed a picture verification task and a picture description task online (Figure 2). The prime sentences were either correct or containing grammar errors, and participants' responses were analyzed to see if their production preferences were changed by the error. The purpose of this experiment was to test three hypotheses, investigating whether ungrammatical sentences would change the production preferences.

### Participants

Ninety participants (35 females, 54 males, 1 did not disclose) were recruited online through Amazon Mechanical Turk, and performed the experiment in exchange for monetary compensation ($15 per hour). Only subjects who identified themselves as native English speakers were allowed to provide norming data. In addition, to obtain high quality data, only MTurk workers with at least a 95% approval rating from previous jobs were included. Ethnicity included 51.1% White, 36.7% Asian, 6.7% African American, 3.3% Latino or Hispanic American, and 2.2% Others. All participants were screened through a pre-experimental survey that gathered information about their language experience and background; only native English speakers without any history of brain damage, reading problems, nor language-related disorder were allowed to proceed to the experiment. One subject was excluded for more than half incomplete or random responses in the language production task. The experimental protocol and inclusion criteria were approved by the Institutional Review Board at the University of Washington.

### Materials

This picture description task was modified based on Hardy et al. (2017)'s experiment. A total of 36 trials with prime target pairs were created. Each picture depicted a transitive action involving an agent and a patient. The verb of the action was printed under each picture. The prime sentence was either active-tense form grammatically correct (AC), passive-tense form grammatically correct (PC), active-tense form grammatically incorrect (AI) or passive-tense form grammatically incorrect (PI). In the total of 36 trials, half (*N* = 18) were Active (A) and the other half were Passive (P); one third of trials (*N* = 12) were grammatically incorrect(I) and two third (*N* = 24) of trials were grammatically correct(I) primes. Because subjects were led to believe that the primes they read were written by the other real participant synchronically, we decided to create less ungrammatical primes than grammatical primes. The order effect was controlled by counterbalancing four prime conditions across lists such that each condition appeared an equal number of times across the experiment, and each item was shown only once.

Ungrammatical prime sentences in the Passive Incorrect syntax condition (PI) were generated using seemingly correct but non-existing past participles modeled after existing verbs, such as “*chasen*” instead of “*chased*,” “*slapt*” instead of “*slapped*,” and “*shooted*” instead of “*shot*.” The ungrammatical verb form was created based on irregular past tense forms; thus, although non-existent, these forms were created using morphosyntactic rules that exist in English. In half of the trials within each condition, the prime picture and prime sentence were perfectly matched, while in the other half, the prime sentence was modified as semantically incorrect by which the identity of either agent or patient is wrong. This manipulation was designed to both make sure that participants were performing the task correctly and to separately measure the effect of syntactic errors from semantic errors.

This study was a 2×2×2 within-subject design, with three of the factors being prime syntax (active vs. passive), grammatical correctness (correct vs. incorrect), and semantic correctness (correct vs. incorrect). In our notation, 4 syntax conditions: AC, AI, PC, PI × 2 semantic conditions: SC (semantically correct) and SI (semantically incorrect). Given that previous studies have demonstrated a stronger syntactic priming effect as prime and target are overlapping (Pickering and Branigan, 1998), in this study, the prime and the target always shared the same action verb. The combination of three independent variable pairs were pseudo-randomized so in each syntax condition (AC, AI, PC, PI), each verb only occurred once, and each verb was modified as both semantic-correct and semantic-incorrect form.

### Procedure

Most SP experiments make use of realistic, in person dialogue between two participants, one of which is a confederate. The confederate verbally utters the primes and the participants' responses are recorded for transcription. To simulate this seemingly realistic dialog situation online, the study described here used deception to convince participants that they were paired with another online “partner” and they were to take turns providing a description for a sentence and verifying the accuracy of their partner's description. In fact, there was no paired partner and all sentences typed by the partner were decided beforehand. At the end of the study, participants were fully debriefed about the use of deception.

In the online task, participants saw a prime picture and were asked to verify whether the sentence constructed by the partner was correctly describing the picture or not. This simple true/false task was created (half true and half false) for two purposes: First, participants were encouraged to attend to the primes; Second, half misplaced trials were used to see whether semantic confusion confounded the syntactic priming effects. Followed by the verification task, there was a picture description task. In the picture description phase, a picture and an appropriate verb were given, and participants needed to type a sentence to describe the picture using the given verb. Participants were told that the game was proceeding in which the partner and the participant alternated between verifying if a sentence-picture pair was matching and constructing a sentence to describe a new picture to the other. The game set a randomly generated waiting time to simulate the amount of time needed by the fictional partner to type their own description.

The participant needed to complete a pre-screen survey that only eligible ones can continue. After giving consent, participants started with a three-trial practice phase to familiarize themselves with the procedure. Between verification task and picture description task, the game set a randomly generated waiting time to simulate the verifying period of the “partner.” At the end of the study, participants were given the debrief about the deception involved and were asked to complete a post-experiment survey.

### Results

**Figure 3:**
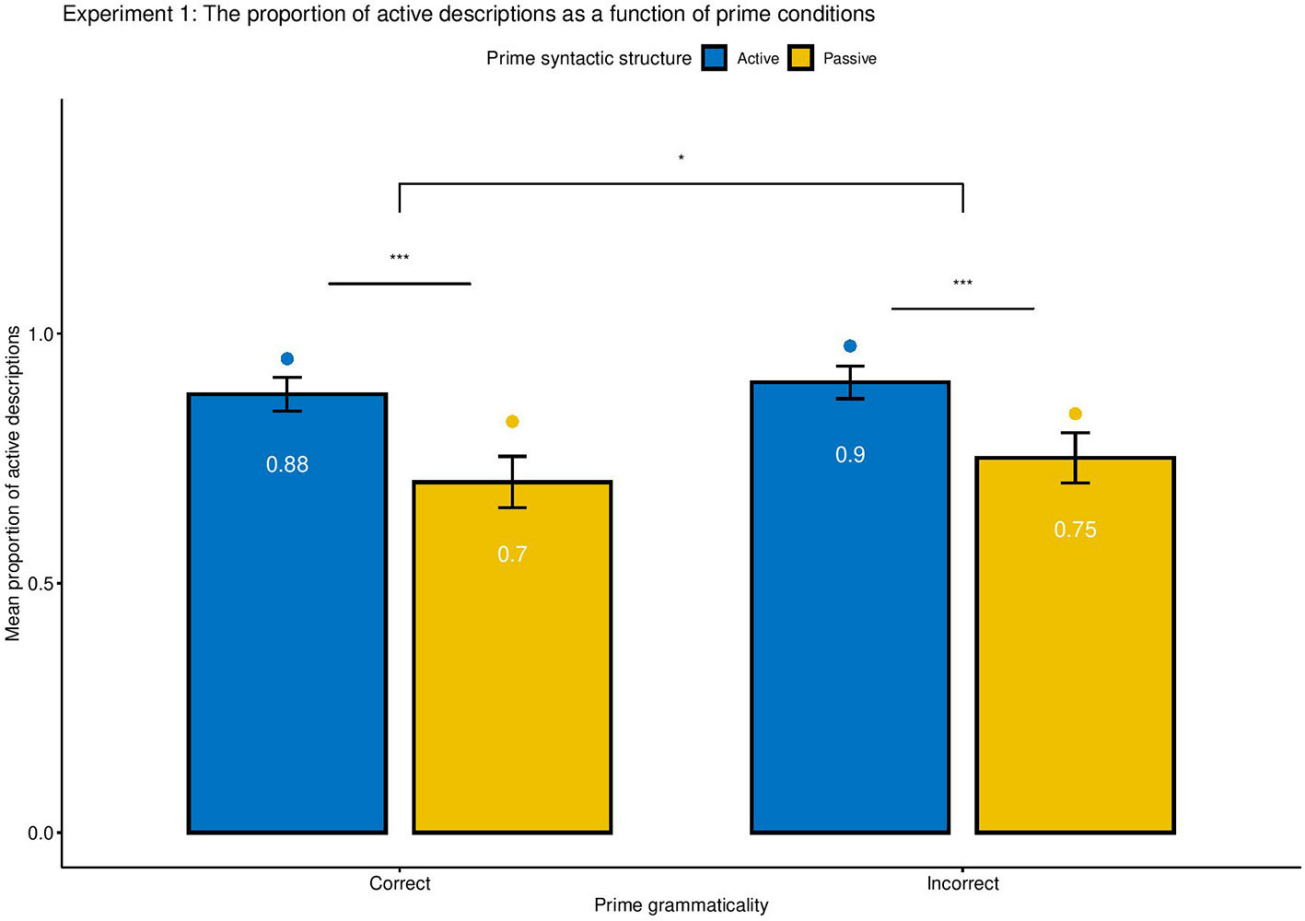
Experiment 1: Mean proportion of producing active constructions as a function of prime grammaticality (Correct vs. Incorrect), syntactic voice (Active vs. Passive). The bar graph and the numbers within each bar represent the mean proportion of produced descriptions across participants, with error bars representing 95% confidence interval. The dot plot represents the estimated proportion transformed by mixed effect logistic model outputs. Asterisk indicates significance level (*** means *p* < 0.001, * indicates *p* < 0.05), and n.s. indicates non-significant results. No interaction between Syntactic Structure and Syntactic Correctness was found, *z* = −0.6, *p* > 0.1. Significant priming effects were found controlling for syntactic correctness and semantic correctness, *p* < 0.01, and there were robust significant effects of grammaticality on the preferences of syntactic constructions, regardless of semantic correctness, *p* < = 0.05.

The syntactic structure of responses typed by participants were first automatically coded with the Natural Language Toolkit package (NLTK; Bird et al., 2009), as Active, Passive or N/A, and the double-checked manually. The total of 3,241 responses yielded 78.74% active-voice and 19.84% passive-voice descriptions. The remaining 1.42% responses, coded as neither active nor passive descriptions, were excluded for further analysis. Small grammar errors and typos (e.g., “A painter punch the monk.”) were included in the analysis as long as their syntactic structure was clearly recognizable, and their grammaticality was coded as either 1 (has error) or 0 (no error). The analysis was conducted with logistic mixed-effects models using orthogonal contrast coding as implemented in the lme4 package in R (as Slevc and Ferreira, 2013). Because the proportion of Active and Passive descriptions are complementary, we only analyzed the proportion of Active sentences produced by each participant in each condition. Syntactic Structure (Active or Passive) and Syntactic correctness (Correct or Incorrect) were treated as fixed effects, and individual subjects were treated as random effects. The parameters were estimated based on the maximum likelihood. Figure 3 demonstrates the mean proportion of active production as a function of prime syntactic structure (Active vs. Passive), prime syntactic correctness (Correct vs. Incorrect). No significant semantic effect was found; thus it is not plotted. For the ease of understanding, the analysis focused on raw proportions rather than log-odds ratios. Following Ivanova et al. (2012), the priming effect was calculated as the proportion of active responses following active primes minus the proportion of active responses following passive primes. Table 3.1 showed the proportion of active responses under various priming conditions. The full statistical results of the logistic mixed effects models were reported in Table 4.1 (as in Slevc and Ferreira, 2013).

**Table 3.1:** Experiment 1 proportion results.

**Prime condition**		**Proportion (Active)**	**Priming effect**
Overall	Active	0.883	0.167
	Passive	0.716	
Overall	Grammatical	0.787	
	Ungrammatical	0.824	
**Two-way interaction comparison**			
Grammatical	Active	0.872	0.170
	Passive	0.702	
Ungrammatical	Active	0.904	0.159
	Passive	0.745	

*1 subject was removed, NA responses were excluded in proportions*.

Overall, syntactic priming effects were observed in written productions. Participants tended to produce 16.7% more active constructions after active than passive prime sentences, regardless of grammaticality, *z* = 8.29, *SE* = 1.43, *p* < 0.001. Specifically, the priming effect of grammatical constructions is 17.0%, *z* = 11.4, *SE* = 0.85, *p* < 0.01, and that of ungrammatical constructions is 15.9%, *z* = 7.61, *SE* = 1.34, *p* < 0.01. There was a significant effect of grammaticality on the preference of syntactic productions. Participants produced 3.7% more active constructions after primed with ungrammatical than grammatical sentences, regardless of syntactic structure, *z* = −2.2, *SE* = 0.11, *p* = 0.028. No significant interaction of syntactic structure and grammaticality was found, *p* > 0.1, suggesting that the SP effect was not mediated by the grammaticality of primes. Moreover, no significant effect of semantics on the preference of syntactic production, *p* > 0.1, eliminating the possible confounding effect of semantic confusions in the tendency of producing particular syntactic structure.

Of interest to our research question, the interaction analyses of grammaticality indicated that specifically for active primes, participants produced 3.2% more active constructions after primed with ungrammatical than grammatical sentences, *z* = −2.30, SE = 0.13, *p* < 0.05, while the pattern was reversed for passive primes, which participants produced 4.3% less passive constructions after primed with ungrammatical than grammatical sentences, *z* = −2.34, *SE* = 0.11, *p* < 0.05.

Overall in this written production task, 8.61% responses were coded as ungrammatical responses. As found in Ivanova et al. (2012), participants tended to make more grammar errors following the ungrammatical primes (*M* = 0.257, *SD* = 0.437) than grammatical primes (*M* = 0.204, *SD* = 0.403), regardless of syntactic structure, *z* = −3.98, *p* < 0.001. No significant effect of semantics was found in the grammaticality of written responses, *p* > 0.1, suggesting that the tendency of making more grammar errors was not enhanced by priming semantic confusions, but enhanced by priming ungrammatical sentences. As for the performance in the semantic verification task, the overall correct verification rate was 79.92%. There was significant effect of semantics on verification rate, with lower verification rate following semantically incorrect primes (*M* = 0.783, *SD* = 0.412) than semantically correct primes (*M* = 0.845, *SD* = 0.362), *z* = −9.09, *p* < 0.001. Moreover, the effect of grammaticality was significant as well, with lower verification rate was found for ungrammatical primes (*M* = 0.720, *SD* = 0.449) than grammatical primes (*M* = 0.860, *SD* = 0.347), *z* = −2.14, *p* < 0.05, Finally, there was a significant effect of syntactic structure on the verification rate, with lower verification rate after passive primes (*M* = 0.791, *SD* = 0.407) than active primes (*M* = 0.837, *SD* = 0.370), *z* = −2.52, *p* < 0.005. These results suggest that sentences that were, for any reason, slightly more difficult to parse (because of passive voice or ungrammatical construction) were also harder to verify. The interaction between semantics, syntactic structure and syntactic correctness was also found significant, *p* < 0.001, suggesting that the performance of verifying semantics was mediated by grammaticality and syntactic structure of the prime.

### Computational Model Analysis

To examine which model accounts for the observed SP pattern better, each model was fit to each participant independently. The log-likelihood of each combination of parameters of each model was calculated by summing up the log-likelihood of obtaining the predicted responses to each of the four types of prime sentences; then, the BIC values of each model parametrization was calculated using (Equation 4). The BIC of each possible parametrization of each model was computed and compared. Given the best fit parameters set for each model, we compared the BIC among three models and the one with minimum BIC has the highest likelihood of fitting empirical data. Although the syntactic priming effect reveals high variability across participants, our models greatly capture the individual differences in Experiment 1.

**Figure 4:**
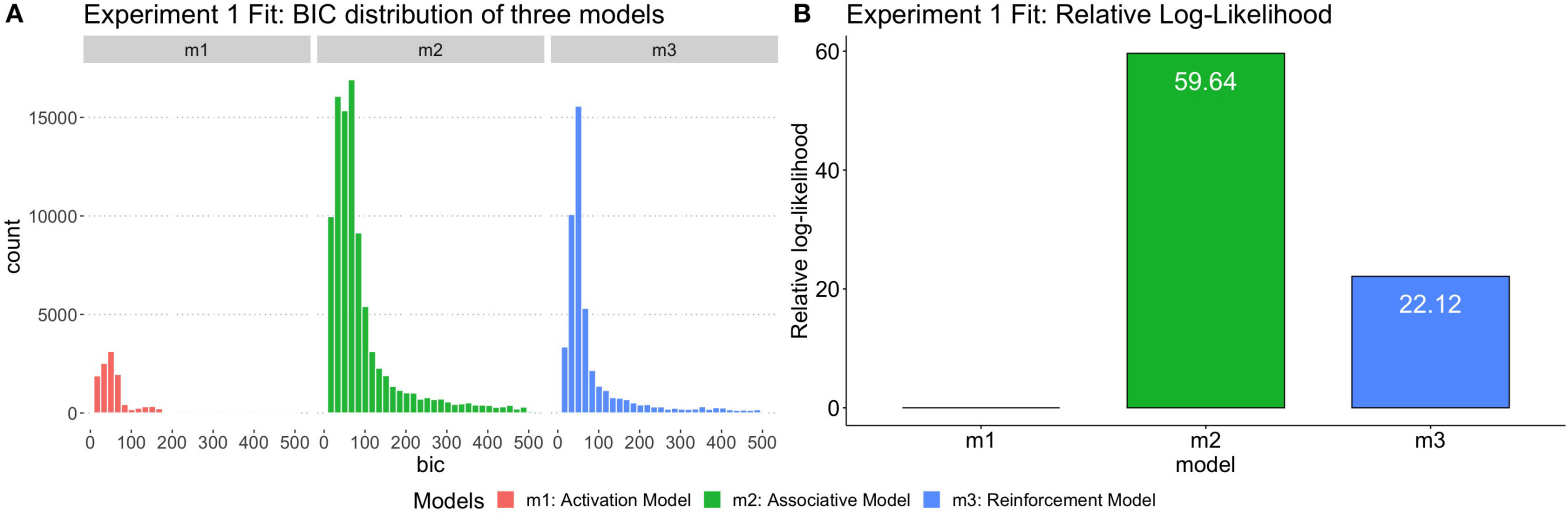
**(A)** The distribution of BIC for three models fit by Experiment 1. **(B)** The relative log-likelihood of three models fit by Experiment 1 data. The relative-likelihood is the ratio of the group-level likelihood to the minimum log-likelihood of three models. Consistent with statistical analysis, one subject was excluded from simulation data analysis as well.

Following the GBF approach, the group-level likelihood value was calculated for each model by summing up the individual likelihoods of the best-fitting parametrization of the model for each participant. The ratio of likelihoods was then compared to yield a Bayes factor as relative-likelihood. Figure 4 shows the BIC distribution across three models and the relative likelihood of three models. Note that the BIC distribution is calculated from the results of each run of the simulations; thus, the Activation model, having a smaller number of parameters, also has a smaller number of observations and a lower distribution which suggests that compared to Reinforcement Model (*m*_3_), Associative Model (*m*_2_) has a higher likelihood of fitting the empirical pattern. According to the Kass and Raftery (1995)'s Bayes Factor interpretation reference, 3.2 < *BF* < 10 means “Substantial” evidence of supporting one model over the base model, 10 < *BF* < 100 means “Strong” evidence, and *BF* > 100 means “Decisive” evidence. As shown in Table 5.1, the Bayes Factor between Associative Model vs. Activation Model (BF[*m*_2_:*m*_1_] = 7.99e+25) was > 100, suggesting “decisive” evidence of supporting the Spreading Model over the Declarative Model. Moreover, the Bayes Factor between Associative Model vs. Reinforcement Model (BF[*m*_2_:*m*_3_] = 1.98e+16) was also > 100, and greater than other BFs. Thus, the group likelihood analysis strongly supported the Associative Model being the best explanation of the behavioral results.

### Discussion

Taken together, the results of our experiment provided a picture that was not entirely consistent with any of the previously discussed models, while the SP was present and robust (albeit less dramatic that in previous studies). Contrary to the predictions of the Activation Model, there was a robust effect of syntactic grammaticality. These effects, however, did not comply precisely with either of the two competing accounts, that is, the Associative and the RL account. In the passive sentences, an ungrammatical prime increased the likelihood of producing another active sentence, consistently. However, the data also showed that semantic errors did not produce any effect, and, therefore, that the effect of errors can be localized to the processes of syntactic parsing.

One possible explanation for the lack of correspondence between the experimental results and our model is that our three models failed to take into account the different ways in which active and passive sentences are parsed. The empirical pattern could be explained by the mixture of base level learning and associative learning accounts. The pattern of Active prime follows hybrid account while the pattern of Passive prime follows the Procedural/RL account. In AI prime, the error was reflected in missing the *s* in the third person singular verb, while in PI prime, the error was reflected in adding seemingly correct but non-existing past participles modeled after existing verbs. Another possible explanation is that our Procedural/RL models are too naive and did not take into account the sequential and incremental nature of error detecting and reward granting. In a previous study (Yang and Stocco, 2019), we proposed a more complex version of the RL-based sequential procedural account, which takes the sequence of parsing into account. In particular, while the naive Procedural/RL model assumed that subjects immediately detected the structure of the sentence (active vs. passive) and generated all feedback signals at the very end of the comprehension process, empirical data suggested that subjects might delay the choice of the correct syntactic form until the first key word was encountered, and generated feedback signals both the end (when all sentences are successfully understood) and as soon as the first incorrect word was found (for ungrammatical ones). This creates a novel asymmetry between the ungrammatical, active (AI) and ungrammatical, passive (PI) sentences. In the case of passive sentences, the firstly encountered verb form is the word “is” [as in “the robber is chased (…)”]; when the word “is” is encountered, under this binary syntactic condition, passive structure is selected with no doubt. The grammatical mistake is then detected immediately thereafter [as in “the robber is chasen (…)”], thus generating a negative feedback that decreases the utility of the passive form. In this condition, therefore, the effect of grammaticality is identical to what was predicted by the previous model. In the case of ungrammatical active sentences, the first verb form is also the first word for which a negative feedback signal can be generated [as in “the robber chase (…)”]. In this case, the negative feedback is generated at the same time or before the active sentence structure is selected, and, thus, does not affect the utility of the corresponding production. When the model successfully completes the sentence comprehension goal, a positive feedback signal is generated that propagates back to active form, thus increasing its utility even if the sentence was ungrammatical.

While the solution proposed in Yang and Stocco (2019) does produce a pattern of results consistent with the observed data, it does so at the expense of adding additional assumptions to the model, which are not easily captured in the complexity penalty of the BIC. Furthermore, it leaves open the question of whether the other two models could also account for the grammaticality effect once their parsing mechanisms are modified.

To avoid these pitfalls, a second experiment was conducted. This experiment was designed so that, in the incorrect sentences, the error would not occur before the syntactic structure could be detected, ruling out the explanation proposed by Yang and Stocco (2019). Second, all of the grammatical errors were created by manipulating the argument structure of the verb, thus avoiding the use of non-words.

## Experiment 2

The purpose of Experiment 2 was to examine the SP pattern with a different syntactic structure alternative. Specifically, we chose the double object construction (DO: “Mary gave John the book”) and the prepositional dative construction (PD: “Mary gave the book to John”). We decided to use DO/PD structure as prime stimulus because unlike the Active/Passive constructions where the grammar error is always attached to the verb, we could manipulate the position of grammar error by introducing the error before the syntactic structure is determined. This avoids the potential confounds of Experiment 1, in which ungrammatical sentences contained morphological violations and non-existing word forms. It also rules out the alternative explanation put forward by Yang and Stocco (2019).

### Participants

One hundred and forty-two University of Washington undergraduates participated in this experiment (84 females, 55 males, 3 did not disclose; mean age 18.8 with SD 1.15). Seventeen participants were excluded in statistical analysis for having more than 4 void responses. Same pre-screen survey was used to collect participant's language background. Only native English speakers were able to proceed. Similar to Experiment 1, ethnicity includes 47.5% White, 41% Asian, 1.44% African American, 0.72% Latino or Hispanic American, and 7.91% Others. The experimental protocol and inclusion criteria were approved by the Institutional Review Board at the University of Washington.

### Materials and Procedure

The paradigm was largely identical to the one used in Experiment 1, but the experiment design was a 2×2 within-subject design, with the factors being prime syntax (double object vs. prepositional dative object), and grammatical correctness (correct vs. incorrect). Because Semantic manipulation had no significant effect in Experiment 1, this factor was not included in this experiment. The stimuli included 20 prime trials and 10 fillers, where one third of trials (*N* = 10) were Dative Object (DO) and one third (*N* = 10) were Prepositional Dative(PD); of the prime trials, half within each condition (*N* = 5, in total *N* = 10) were grammatically correct (C) and the other half of them were grammatically incorrect (I). The prime sentence depicted a transitive action involving an agent and a patient such as *hand, give, show*. The filler trial depicted intransitive actions such as *nap, brush, wash (e.g., The cat is napping on the windowsill)*. The verb of the action was printed under each target picture. The prime sentence was either grammatically correct DO form (DOC: *e.g., The man handed the clown a hat*), grammatically correct prepositional-dative form (PDC: *e.g., The swimmer handed the towel to the driver*), grammatically incorrect double-object-dative form (DOI: e.g., *The captain gave the old sailor they spare life jacket*) or grammatically incorrect PD form (PDI: *e.g., The builder showed the blueprints to them new client*). The order of trials was pseudorandomized so that each filler was between a block of 4 different prime conditions.

In order to have valid and natural ungrammatical sentences, we pretested the stimuli by inserting different grammar errors into sentences in a pilot study. Another group of subjects were recruited to rate the sentence errors. Grammar errors that elicited both semantic and syntactic confusions were not used in Experiment 2. Pilot study revealed that whether it was realistic, in person dialogue did not greatly change the way people produce sentences in this scenario, thus in Experiment 2, we did not instruct participants to communicate with a confederate. They only needed to judge whether the prime sentence is grammatically correct or not, and then to describe the target picture using the verb provided. This simple verification task, similar to the verification task in Experiment 1, was used to encourage participants to attend to the prime sentences, and assess whether the grammar error could be detected effectively.

### Results

**Figure 5:**
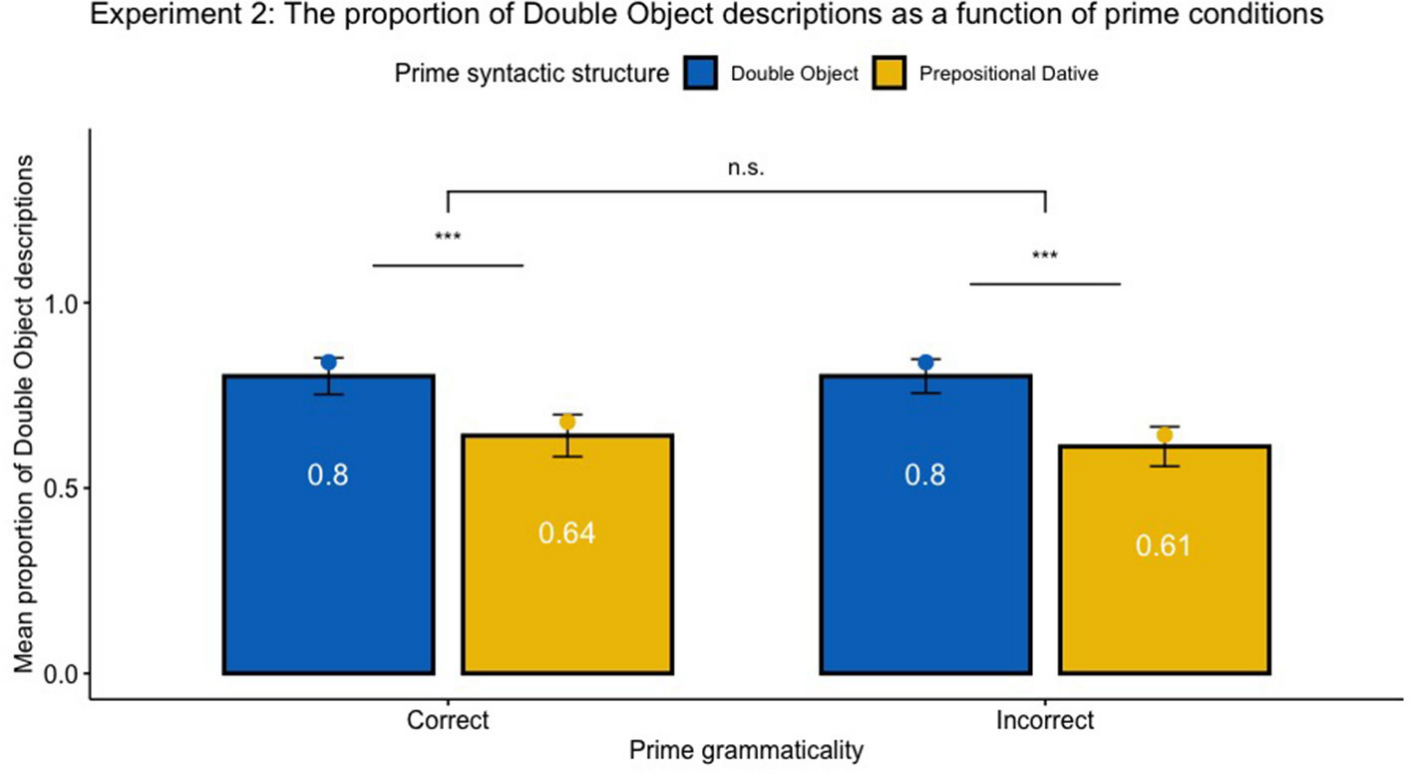
Experiment 2: Mean proportion of producing DO constructions as a function of prime grammaticality (Correct vs. Incorrect) and syntactic structure (DO vs. PD). The bar graph and the numbers within each bar represent the mean proportion of produced descriptions across participants, with error bars representing 95% confidence interval. The dot plot represents the estimated proportion transformed by mixed effect logistic model outputs. Asterisk indicates significance level (*** means *p* < 0.001, and n.s. indicates non-significant results). No significant effect of grammaticality was found, *z* = 1.2, *p* > 0.1. No interaction between Syntactic Structure and Syntactic Correctness was found, *z* = −0.76, *p* > 0.1. Statistical analysis showed significant priming effects, controlling for syntactic correctness, *p* < 0.01.

The syntactic structure of responses typed by participants were first automatically coded with the Natural Language Toolkit package (NLTK; Bird et al., 2009), as Dative Object (DO), Prepositional dative (PD) or N/A, and then double-checked manually. The total of 2,840 responses yielded 64.47% DO, and 28.06% PD descriptions. The remaining 7.46% responses coded as neither DO nor PD constructions were excluded for further analysis. Similar to Experiment 1, responses with minor grammatical errors and typos (e.g., “The sailor gives the an a teapot”) were included in the analysis, and the grammaticality of responses was coded as 1 (error) or 0 (no error). The analysis was conducted with logistic mixed-effects models using orthogonal contrast coding as implemented in the lme4 package in R (as Slevc and Ferreira, 2013). Because the proportion of DO and PD descriptions are complementary, we only analyzed the proportion of DO sentences produced by each participant in each condition. Syntactic Structure (DO or PD) and Syntactic correctness (Correct or Incorrect) were treated as fixed effects, and individual subjects were treated as random effects. The parameters were estimated based on the maximum likelihood. Similar to Experiment 1, the analysis focused on raw proportions rather than log-odds ratios. The priming effect was calculated as the proportion of DO responses following DO primes minus the proportion of DO responses following PD primes. Figure 5 shows the mean proportion of DO descriptions as a function of prime syntactic structure (DO vs. PD), prime syntactic correctness (Correct vs. Incorrect). Table 4.2 showed the proportion of DO responses under various priming conditions. Table 3.2 shows the full statistical results of the logistic mixed effects models.

**Table 3.2:** Logistic fixed effects model coefficients and statistical tests from experiment 2.

**Statistical test**		**Odds ratios**	***SE***	***z***	***p***
**Syntactic priming**					
	(Intercept)	1.80^***^	0.26	4.07	**<0.001**
	Syntactic Structure[DO]	2.90^***^	0.42	7.29	**<0.001**
	Syntactic Correctness[C]	1.17	0.16	1.2	0.229
	Syntactic Structure[DO]: Syntactic Correctness [C]	0.86	0.17	−0.76	0.447
	**Random Effects**				
	R2	3.29			
	ICC	0.31			
	N surveyID	125			
	Observations	2,429			
	Marginal R2/Conditional R2	0.049/0.345			
	log-Likelihood	−1274.984			
	**Simple main effects, where prime conditio*****n =*** **grammatical**				
	(Intercept)	2.15^***^	0.34	4.86	**<0.001**
	Syntactic Structure[DO]	2.57^***^	0.38	6.33	**<0.001**
	**Simple main effects, where prime conditio*****n =*** **ungrammatical**				
	(Intercept)	1.77^***^	0.24	4.27	**<0.001**
	Syntactic Structure[A]	2.76^***^	0.40	7.06	**<0.001**
**Interaction analysis of grammaticality**					
	**prime conditio*****n =*** **DO**				
	(Intercept)	5.38^***^	0.93	9.75	**<0.001**
	Syntactic Correctness[C]	1.01	0.16	0.07	0.944
	**prime conditio*****n =*** **PD**				
	(Intercept)	1.86^***^	1.38–2.50	4.05	**<0.001**
	Syntactic Correctness[C]	1.18	0.16	1.23	0.217
**Analysis of production error**					
	(Intercept)	154.21^***^	64.33	12.08	<0.001
	Syntactic Structure[DO]	0.62	0.22	−1.35	0.178
	Syntactic Correctness[C]	0.56	0.19	−1.66	0.097
	Syntactic Structure[DO]: Syntactic Correctness[C]	3.83^**^	1.96	2.62	0.009

*NA responses were excluded in analysis*.

*p < 0.05;

** *p < 0.01;*

*** *p < 0.001*.

**Table 4.1:** Logistic fixed effects model coefficients and statistical tests from experiment 1.

**Statistical test**		**Odds ratios**	***SE***	***z***	***p***
**Syntactic priming**					
	(Intercept)	5.71^***^	1.62	6.16	**<0.001**
	Syntactic Structure[A]	6.40^***^	1.43	8.29	**<0.001**
	Syntactic Correctness[C]	0.72^*^	0.11	−2.2	**0.028**
	Semantic Correctness[C]	1.18	0.13	1.47	0.142
	Syntactic Structure Syntactic Correctness	0.86	0.22	−0.6	0.547
	**Random effects**				
	R2	3.29			
	ICC	0.6			
	N subjID	89			
	Observations	3,179			
	Marginal R2/Conditional R2	0.090/0.638			
	log-Likelihood	−1070.286			
	**Simple main effects, where prime conditio*****n =*** **grammatical**				
	(Intercept)	4.24^***^	1.1	5.59	**<0.001**
	Syntactic Structure[A]	5.60^***^	0.85	11.4	**<0.001**
	**Simple main effects, where prime conditio*****n =*** **ungrammatical**				
	(Intercept)	6.11^***^	1.79	6.17	**<0.001**
	Syntactic Structure[A]	5.80^***^	1.34	7.61	**<0.001**
**Interaction analysis of grammaticality**					
	**prime conditio*****n =*** **Active**				
	(Intercept)	45.89^***^	18.42	9.53	**<0.001**
	Syntactic Correctness[C]	0.61^*^	0.13	−2.3	**0.022**
	**prime conditio*****n =*** **Passive**				
	(Intercept)	6.51^***^	1.9	6.43	**<0.001**
	Syntactic Correctness[C]	0.70^*^	0.11	−2.34	**0.019**
**Analysis of verification rate**					
	(Intercept)	8.10^***^	2.07	8.18	**<0.001**
	Syntactic Structure[A]	0.54^*^	0.13	−2.52	**0.012**
	Syntactic Correctness[C]	0.62^*^	0.14	−2.14	**0.032**
	Semantic Correctness[C]	0.11^***^	0.03	−9.09	**<0.001**
	Syntactic Structure[A]: Syntactic Correctness[C]	1.90^*^	0.57	2.15	**0.032**
	Syntactic Structure[A]: Semantic Correctness[C]	12.42^***^	4.21	7.43	**<0.001**
	Syntactic Correctness[C]: Semantic Correctness[C]	55.92^***^	18.95	11.87	**<0.001**
	Syntactic Structure[A]: Syntactic Correctness[C]: Semantic Correctness[C]	0.19^***^	0.09	−3.31	**0.001**
**Analysis of production error**					
	(Intercept)	0.00^***^	0	−5.43	**<0.001**
	Syntactic Structure[A]	1.14	0.3	0.51	0.609
	Syntactic Correctness[C]	0.40^***^	0.09	−3.98	**<0.001**
	Semantic Correctness[C]	0.92	0.14	−0.5	0.615
	Syntactic Structure[A]: Syntactic Correctness[C]	0.91	0.3	−0.29	0.773

*NA responses were excluded in analysis*.

* *p < 0.05;*

** p < 0.01;

*** *p < 0.001*.

**Table 4.2:** Experiment 2 proportion results.

**Prime condition**		**Proportion (DO)**	**Priming effect**
Overall	DO	0.787	0.159
	PD	0.628	
Overall	Grammatical	0.715	
	Ungrammatical	0.698	
**Two-way interaction comparison**			
Grammatical	DO	0.788	0.145
	PD	0.643	
Ungrammatical	DO	0.786	0.173
	PD	0.614	

*17 subjects were removed, NA responses were excluded in proportions*.

Overall, syntactic priming effects were observed in written productions. Participants tended to produce 15.9% more DO constructions after DO than PD primes, regardless of grammaticality, *z* = 7.29, *SE* = 0.42, *p* < 0.001. Specifically, the priming effect of grammatical constructions is 14.5%, *z* = 6.33, *SE* = 0.38, *p* < 0.001, and that of ungrammatical constructions is 17.3%, *z* = 7.06, *SE* = 0.40, *p* < 0.001. Different from Experiment 1, the effect of grammaticality on the preference of syntactic productions was not significant, *p* > 0.1. No significant interaction of syntactic structure and grammaticality was found, *p* > 0.1, suggesting that the SP effect was not mediated by the grammaticality of primes. The interaction analysis of grammaticality indicated that there was no significant difference of target constructions after primed with ungrammatical sentences than grammatical sentences, *p* > 0.1. Of all responses, 2.64% responses were coded as ungrammatical responses. Unlike Experiment 1, no significant effect of grammaticality of prime on the grammaticality of written responses was found, *p* > 0.05, while the interaction between grammaticality and syntactic structure was significant, *z* = 2.62, *SE* = 1.96, *p* = 0.009, suggesting that the tendency of making more grammar errors was mediated by both grammaticality and syntactic structure of the prime. The following sections will discuss several possible interpretations of the divergent findings between experiment 1 and experiment 2.

### Computational Model Analysis

**Figure 6:**
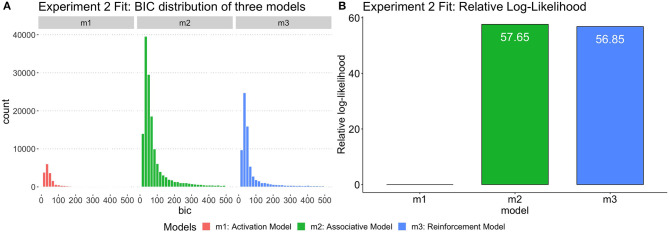
**(A)** The distribution of BIC for three models fit by Experiment 2 data. **(B)** The relative log-likelihood of three models fit by Experiment 2 data. The relative-likelihood is the ratio of the group-level likelihood to the minimum log-likelihood of three models. Consistent with statistical analysis, 17 subjects were excluded from simulation data analysis.

As in Experiment 1, three different models were fit to each participant of experiment 2. In order to evaluate the complexity and goodness-of-fit of three models, all possible simulation outputs were fit into aggregated individual participant data. Similar to the model analysis in Experiment 1, the group-level likelihood was calculated for each model by summing up the individual likelihoods of the best-fitting parametrization of the model for each participant in Experiment 2. The ratio of likelihoods was then compared to yield a Bayes factor as relative-likelihood. Figure 6 showed the BIC distribution across three models and the relative likelihood. The Associative Model has slightly higher relative-likelihood than the Reinforcement Model, which adds further evidence supporting the Associative Model. In Table 5.2, the Bayes Factor of Spreading Mode vs. Activation Model (BF[*m*_2_:*m*_1_]) is > 100, much higher than other BFs in its row. According to the Kass and Raftery (1995)'s Bayes Factor interpretation reference, the Associative Model being the best explanation of the behavioral results. In sum, the simulation results of individual model fitting and group-level BF analysis suggest that an associative account with a declarative representation of syntactic rules is the best theory to explain the empirical data in Experiment 2.

**Table 5.1:** Summary of log-likelihood and BF statistics for experiment 1.

	**Group Log-Likelihood**	**BF[m: m1]**	**BF[m: m2]**	**BF[m: m3]**
Activation Model (m1)	−1277.305	1	1.25E−26	2.48E−10
Associative Model (m2)	−1217.662	7.99E+25	1	1.98E+16
Reinforcement Model (m3)	−1255.188	4.03E+09	5.04E−17	1

**Table 5.2:** Summary of log-likelihood and BF statistics for experiment 2.

	**Group Log-Likelihood**	**BF[m: m1]**	**BF[m: m2]**	**BF[m: m3]**
Activation Model (m1)	−1433.287	1	9.16E−26	2.05E−25
Associative Model (m2)	−1375.635	1.09E+25	1	2.24E+00
Reinforcement Model (m3)	−1376.439	4.88E+24	4.48E−01	1

### Discussion

The priming effect was observed in DO/PD structure, regardless of the grammatical correctness. However, the effect of grammar error seems to be much smaller compared to Experiment 1, being not significant for both PD primes. There are several possibilities to explain this pattern. First, it is possible that there is no effect of grammaticality, suggesting that the null hypothesis is the best account to explain the empirical findings. The priming of ungrammatical constructions is reduced. This would, however, be at odds with the significant effects found in Experiment 1 using active/passive primes. Another possible explanation is that there is no single mechanism of language processing which could be applied to everyone, especially in ungrammatical SP effects. Different people depend on different mechanisms to process grammar errors, which is supported by the great individual differences observed.

A third possibility is that an effect of grammaticality on primes exists, but our results were underpowered and the variability in our participants' performance are partially obscuring the result. This possibility is supported by the modeling approach, which show that, while the overall pattern resembles the prediction of the Activation model, individual participants are almost never fit by it, producing instead results more compatible with the other two models When comparing the models in terms of GBF, the Associative Model, much like in Experiment 1, provides the best account for the data, implying that the patterns of results observed in individual participants, however noisy, do show an effect of surprise.

## General Discussion

This paper has presented the results of two experiments in which the nature of syntactic representations was investigated using the syntactic priming effect. As demonstrated in many syntactic priming studies, people tend to reuse the same syntactic structures they are primed with. Consistent with this body of literature, our experiment showed an overall syntactic priming effect for active and passive structures, regardless of grammatical correctness and semantic correctness. This implies that the tendency of reproducing primed syntactic structures persists even if the priming linguistic structure is erroneous. Three different theories were tested; each theory was implemented as a computational model within the same, general, cognitive architecture, and their predictions were compared to the experimental results.

In both experiments, we found that the empirical group-level pattern of results did not precisely follow the exact predictions of any model. In particular, the effect of grammaticality in Experiment 1 followed partially the predictions of the Associative Model and partially those of the Reinforcement model; in Experiment 2, no effect of grammaticality was found. The difference in findings between the two experiments could potentially be accounted for by the fact that the strong but unexpected grammaticality effect in Experiment 1 was partially due to plausible but non-existent verb forms, and that the grammatical structure could be guessed before the error was detected, leaving an opportunity for different parsing strategies. Experiment 2, which removed both confounds, offers a pattern that is in line with the Activation Model. The group-level finding, however, obscurses significant variability in the data; while at the group level no effect of grammaticality is found, individual participants consistently show effects of grammaticality on priming, suggesting that the group average results is a particular case of “averaging over methods” (Newell, 1973) leading to false results.

This conclusion is supported by a careful model-based analysis of individual subject data. As three models are parametrized to fit each individual, the Associative offers a better fit to individuals than either of the other two models, in terms of Bayes Factor. This finding is consistent across both experiments, as is the finding that the Activation model rarely matches the results of any participant, despite its predictions resembling the group averages. We believe that this approach, in which an individual is matched to a corresponding model, has greater explanatory power as it accounts for both individual differences and explicit comparisons of different hypotheses.

If our modeling conclusions are to be believed, they would imply that syntactic structures are likely represented declaratively, activated by learned associations, and affected by frequency and recency rather than by feedback signals.

This conclusion is perfectly in line with Reitter et al. (2011) model of syntactic priming, which served as the inspiration for our Associative model. It is also consistent with the results of Ivanova et al. (2012), which strongly imply that syntactic structures are represented in a lexicalized, declarative way. And, finally, it is consistent with other ACT-R models that also used declarative knowledge to represent syntactic structures (as in the case of Stocco and Crescentini's 2005 model of aphasia). At the same time, this conclusion seems apparently at odds with Ullman's influential framework (2004), which assumes that all syntactic operations are procedural, and with the mounting evidence for a corresponding role of the basal ganglia in supporting linguistic processes (Lieberman, 2002; Crinion et al., 2006; Kotz et al., 2009; Stocco and Prat, 2014). However, note that, as implied by Ullman's framework, all of our models do contain a mixture of declarative and procedural knowledge. In all models, for example, lexical information is represented declaratively, and all the steps in the parsing process are represented procedurally. In the Associative model, it is only the specific *representation* of syntactic structure that is represented declaratively, rather than procedurally; procedural knowledge is still needed to process and operate on them. Therefore, we maintain that results are still compatible with Ullman's (2004) framework at large (and with a role of subcortical structures in language) even if they reject a stronger version of it.

Still, our results should be considered in light of a number of limitations. First, they do not cover the range of possible syntax structures that could be primed, or grammatical errors that could be induced. Second, it would have been desirable to have a greater sample size, especially since some effects were barely on the threshold of statistical significance. Finally, it is possible that in-person manipulations of syntactic priming during error would have elicited a stronger effect. Given that nowadays the way people communicate is no longer limited to in-person verbal communication, it is important to investigate online typing-based communication. Future studies could examine whether people's way of communication would be different when they realize that the person they speak to is not a real human.

These limitations notwithstanding, we believe that our results do contribute in many ways to the growing body of research on the computations underlying language processing. First, although different hypotheses have been put forward about the nature of SP, our study is the first one to fully compare three different mechanisms. Second, our results highlight the role of prediction and of basic attention mechanisms in language, whose contribution might shed light on the basic computations underlying syntactic parsing in an emergentist fashion (Hernandez et al., 2019). Third, our results highlight the power allowed by using detailed computational models to explain psycholinguistic effects and the importance of analyzing individual-level models in examining theories.

## Data Availability Statement

The datasets for this study can be found in the Github repository: Syntactic Priming https://github.com/UWCCDL/SyntaxPriming.

## Ethics Statement

The studies involving human participants were reviewed and approved by Institutional Review Board, Human Subjects Department, University of Washington. The patients/participants provided their written informed consent to participate in this study.

## Author Contributions

YY and AS contributed to conception, design of the study, wrote the first draft of the manuscript, and contributed to manuscript revision, read, and approved the submitted version. AK contributed to the design and stimuli preparation of the second experiment. YY organized the database, performed the statistical analysis, and completed the coding and analysis. AS provided conception of computational modeling. All authors contributed to the article and approved the submitted version.

## Conflict of Interest

The authors declare that the research was conducted in the absence of any commercial or financial relationships that could be construed as a potential conflict of interest.
